# Composite Cold-Formed Steel Beams with Diagonal Rebars for Earthquake-Resistant Buildings

**DOI:** 10.3390/ma16083002

**Published:** 2023-04-10

**Authors:** James Samuel, Shalini Ramachandran Nair, Philip Saratha Joanna, Beulah Gnana Ananthi Gurupatham, Krishanu Roy, James Boon Piang Lim

**Affiliations:** 1 Department of Civil Engineering, Hindustan Institute of Technology and Science, Padur, Chennai 603103, India; jsamuel@hindustanuniv.ac.in (J.S.);; 2 Division of Structural Engineering, College of Engineering Guindy Campus, Anna University, Chennai 600025, India; 3 School of Engineering, The University of Waikato, Hamilton 3216, New Zealand; 4 Department of Civil and Environmental Engineering, The University of Auckland, Auckland 1023, New Zealand

**Keywords:** cold-formed steel, diagonal web rebars, fly-ash, concrete encasement, ductility, moment–curvature relationship, lateral stiffness, finite element analysis

## Abstract

The construction industry is on the lookout for cost-effective structural members that are also environmentally friendly. Built-up cold-formed steel (CFS) sections with minimal thickness can be used to make beams at a lower cost. Plate buckling in CFS beams with thin webs can be avoided by using thick webs, adding stiffeners, or strengthening the web with diagonal rebars. When CFS beams are designed to carry heavy loads, their depth logically increases, resulting in an increase in building floor height. The experimental and numerical investigation of CFS composite beams reinforced with diagonal web rebars is presented in this paper. A total of twelve built-up CFS beams were used for testing, with the first six designed without web encasement and the remaining six designed with web encasement. The first six were constructed with diagonal rebars in the shear and flexure zones, while the other two with diagonal rebars in the shear zone, and the last two without diagonal rebars. The next set of six beams was constructed in the same manner, but with a concrete encasement of the web, and all the beams were then tested. Fly ash, a pozzolanic waste byproduct of thermal power plants, was used as a 40% replacement for cement in making the test specimens. CFS beam failure characteristics, load–deflection behavior, ductility, load–strain relationship, moment–curvature relationship, and lateral stiffness were all investigated. The results of the experimental tests and the nonlinear finite element analysis performed in ANSYS software were found to be in good agreement. It was discovered that CFS beams with fly ash concrete encased webs have twice the moment resisting capacity of plain CFS beams, resulting in a reduction in building floor height. The results also confirmed that the composite CFS beams have high ductility, making them a reliable choice for earthquake-resistant structures.

## 1. Introduction

Cold-formed steel (CFS) sections are becoming popular for low- to medium-rise construction due to their lower cost and lighter weight [1,2,3]. In the construction industry, sustainable materials are in high demand. Cold-formed steel (CFS) and fly-ash concrete are both environmentally friendly materials, and their combination results in increased load-carrying capacity, good ductility, high fire resistance, a lower rate of corrosion, increased serviceability, and aesthetic appeal [4,5,6].

There has been little research on composite CFS beams. In CFS beams, the addition of a triangular corrugated web increases out-of-plane buckling and overall strength compared to the plain web for the same height/thickness ratio. Corrugations in the web significantly improve its performance, according to experimental and finite element studies [7]. CFS beams with optimised folded-flange sections provided maximum flexural performance at the lowest cost per unit of material [8]. Premature distortional and local buckling failures in built-up CFS beams were eliminated by using intermediate stiffeners with indents [9].

In terms of connections, spot welding was favoured over screw connections for fabricating built-up CFS beams due to its higher bending capacity under flexural loading and sustained beam stiffness. Spot-welded specimens also had greater ductility and tensile strength than screw-connected sections [10]. Screw spacing in built-up CFS members should be reduced to prevent lateral-torsional buckling. When fastener spacing is reduced for sections connected back-to-back, flexural capacity increases [11].

Beams with a shear transfer mechanism demonstrated greater strength and less deflection than beams relying on the natural bond between concrete and steel to resist shear. When compared to beams without a shear transfer mechanism, beams with a shear transfer mechanism had a significant reduction in deflection and a higher shear strength. Shear connectors significantly increased the effectiveness of shear transfer to achieve full composite action [12,13]. The ultimate strength of a short-to medium-span composite beam was governed by shear connectors, whereas the ultimate strength of a medium-to long-span composite beam was governed by the mid-span compressive strength of concrete [14].

The addition of fly ash to concrete as a partial replacement for cement increases matrix density, durability, and sustainability [15,16]. Fly ash concrete beams were found to have good ductility, withstand large displacements, and dissipate seismic energy well [17]. At the manufacturing stage, substituting 50% fly ash for cement increased workability while decreasing the water–cement ratio. The flexural strength, Young’s modulus, and split tensile strength of post-concrete beams remained the same as those of ordinary concrete beams [18]. Compressive strength was achieved more slowly in concrete beams made with 70% fly ash as a replacement for cement, but the crack mechanism, flexural, and split tensile strengths were the same as in conventional concrete beams [19]. For the best performance of concrete beams, the cement replacement by fly ash concrete in the mix should be less than 50% [20]. In compressive strength and chloride penetration tests for compression members, fly ash replacement of cement up to 60% yielded good results. The ideal fly ash content for long-lasting concrete is 60% [21]. Fly-ash concrete beams cured for 56 days performed the same as ordinary Portland cement beams cured for 28 days. Cracks appeared in fly-ash concrete beams within the limits prescribed by the IS 456-2000 code [22]. The performance of beams with up to 40% cement replacement in fly ash was identical to that of RC mixes without fly ash [23].

Concrete and steel can be joined to form a composite structure that functions as a single unit. The composite steel–concrete structure was stronger and stiffer than the steel or concrete counterparts [24,25,26,27,28,29,30]. Composite CFS–concrete composite beams had good bending strength, a high rotation capacity, and less deformation [31]. The use of concrete on both sides of the web saves materials in beams. The beam was given overall stability by preventing local buckling of the slender steel web by using concrete on both sides. Shear connectors [32] enabled load transfer from the web to concrete. Composite beams outperform steel or reinforced concrete equivalents in terms of strength-to-weight ratio and ductility index. Lips were found to increase load-carrying capacity in the tension zone of composite beams. The composite beams failed gradually, with steel yielding, local buckling, slip between concrete and steel, concrete cracking, and final separation of two materials [33]. Composite CFS beams outperformed rolled steel sections in terms of strength-to-weight ratio. Material design strengths were greater when material and geometrical imperfections were present [34].

The use of spacers in open sections prevents distortional buckling failure by increasing its torsional strength [35]. For built-up sections, the finite element analysis produced more accurate results than the direct strength method. Members’ flexural performance was improved by built-up sections with complex lips. When beam and shell elements were used to accurately represent shear connectors in FE modelling, premature failure of shear connectors was avoided [36]. The structurally intuitive FE models allowed for various load combinations and boundary conditions [37].

Through the use of an effective connector arrangement that prevented early slip between CFS and concrete, CFS and concrete beams subjected to composite action could be designed to fail because of issues related to ductility. Flexural strength predictions were correct for composite beams that experienced ductile failure [38]. Beams with confinement demonstrated high energy absorption capacity and ductility, making them suitable for use in earthquake-resistant construction [39].

However, it is to be mentioned that no research has been conducted on CFS beams with diagonal rebars in the web and the web encased in concrete. The mode of failure, moment capacity, lateral stiffness, and ductility of CFS beams with diagonal rebars and web encased in fly ash concrete were investigated in this study. The results of the tests were also compared to the numerical results.

## 2. Materials and Methods

### 2.1. Cold-Formed Steel and Rebars

The required dimensions of built-up CFS beams were fabricated from readily available steel sheets measuring 2.4 m by 1.2 m and having a thickness of 2 mm. Tensile tests were carried out on coupons taken from the same CFS sheets in accordance with the ASTM A370 standard [40].

The ultimate tensile strength of the cold-formed steel used for the specimens was 450 N/mm^2^, and the yield strength was 350 N/mm^2^. Rebars of 6 mm diameter were used as diagonals in the web. The ultimate strength and yield strength of rebars used as diagonals were 490 N/mm^2^ and 415 N/mm^2^, respectively.

### 2.2. Fly-Ash Concrete

By replacing ordinary Portland cement (OPC) with 40% high-volume fly ash, an eco-friendly concrete was developed. Crushed stones of 4.75 mm size, also known as manufactured sand (M-sand), were used as fine aggregate; stones of 20 mm and 12 mm in a 60:40 ratio were used as coarse aggregates; 0.7% Glenium Master Sky 8233 (manufacturer: BASF Glenium Master Sky 8233, Chennai, India) was used as a superplasticizer; and 1% Polyethylene Glycol 600 (manufacturer: Fisher Scientific, Mumbai, India) was used as a self-curing agent. For M30-grade concrete, the mix proportion was 1:1.55:3.16, with a water/cement ratio of 0.4.

### 2.3. Fabrication of CFS Beams

The beam was 2 m long with a cross-section of 100 mm × 150 mm × 2 mm. Using a press braking machine, the two edges of the flange were bent 90° to form 10 mm lips. Intermittent fillet welding was used to connect the flanges and webs to form the I-section. To stiffen the web against out-of-plane buckling, stiffener plates of the same material and thickness were cut and welded to it at the loading and reaction points. Rebars of 6 mm diameter were welded diagonally on both sides of the webs in the shear and flexure zones as needed. M30-grade fly ash concrete was used to encase both sides of the web.

**Figure 1:**
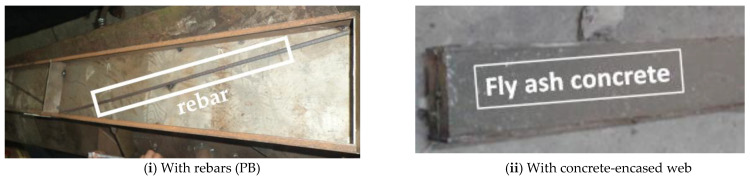
Fabrication of CFS beams with rebars and concrete-encased webs.

**Figure 2:**
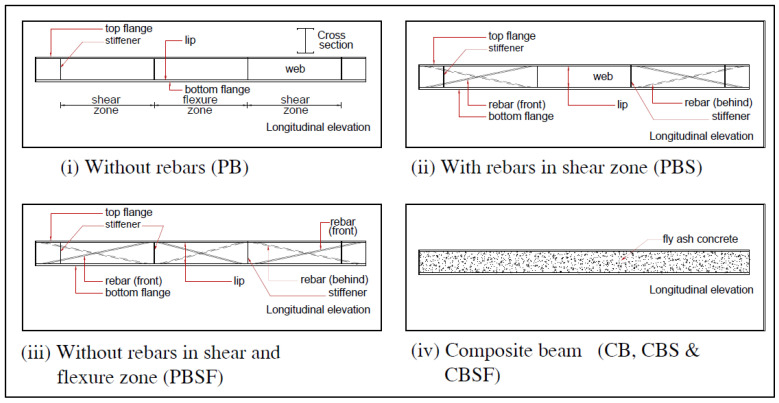
Schematic diagrams of CFS beams.

The fabrication of a CFS beam with rebars and a concrete-encased web is shown in Figure 1. Figure 2i–iv depict schematic diagrams of plain CFS beams without diagonal rebars, diagonal rebar in the shear zone, diagonal rebar in the shear and flexure zones, and composite CFS beams.

## 3. Experimental Investigation

### 3.1. Specimen Details

Plain CFS beams were tested with and without diagonal rebars in the web (PB), diagonal rebars in the shear zone (PBS), diagonal rebars in the shear and flexure zones (PBSF), and web encased with fly-ash concrete (CB, CBS, and CBSF). Table 1 lists the characteristics of the tested beams.

### 3.2. Test Set-Up

**Figure 3:**
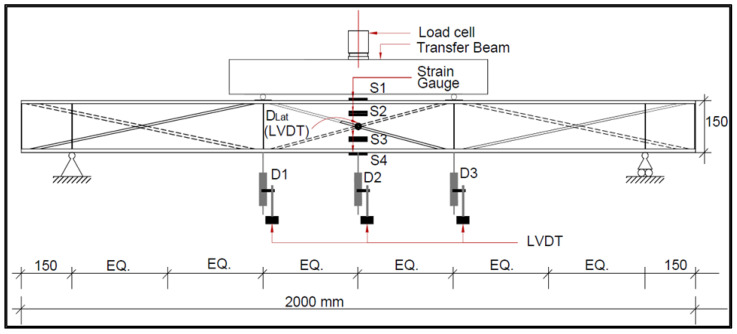
Schematic diagram of the experimental setup.

All of the beams were tested in a vertical loading frame under two-point loading conditions. The beams were tested as simply supported beams with a span of 1700 mm and two-point loads at one-third the distance from the supports. The load cell was used near the central load, which was divided into two equal point loads using the transfer beam, and the load was gradually increased at a rate of 2 kN/min until the specimen failed. Figure 3 depicts a schematic diagram of the experimental setup.

The vertical deflections of the beam were measured using three LVDTs (D1, D2, and D3) placed at mid-span and below the two-point loads. An LVDT (DLat) was placed horizontally in the centre to capture the lateral deflection. To record the strains, strain gauges (S1, S2, S3, and S4) were also attached to the beam. The data logger was linked to the load cell, strain gauges, and LVDTs.

## 4. Results and Discussion

### 4.1. Failure Modes

**Figure 4:**
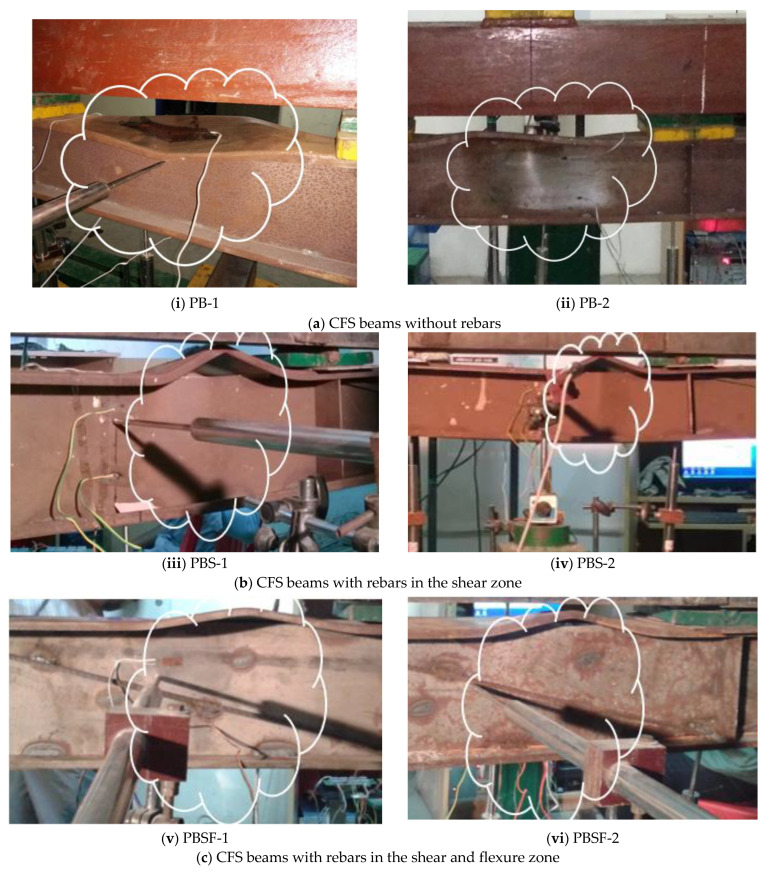
Photograph of the CFS beams at failure.

Plain CFS beams without rebars in the web (PB) and with rebars in the shear zones of the web (PBS) failed at the centre of the span due to local buckling of the top compression flange and web buckling over the full depth. The beams with rebars in the shear and flexure zones of the web (PBSF) failed due to top flange local buckling and web buckling only at the top compression flange junction (Figure 4).

**Figure 5:**
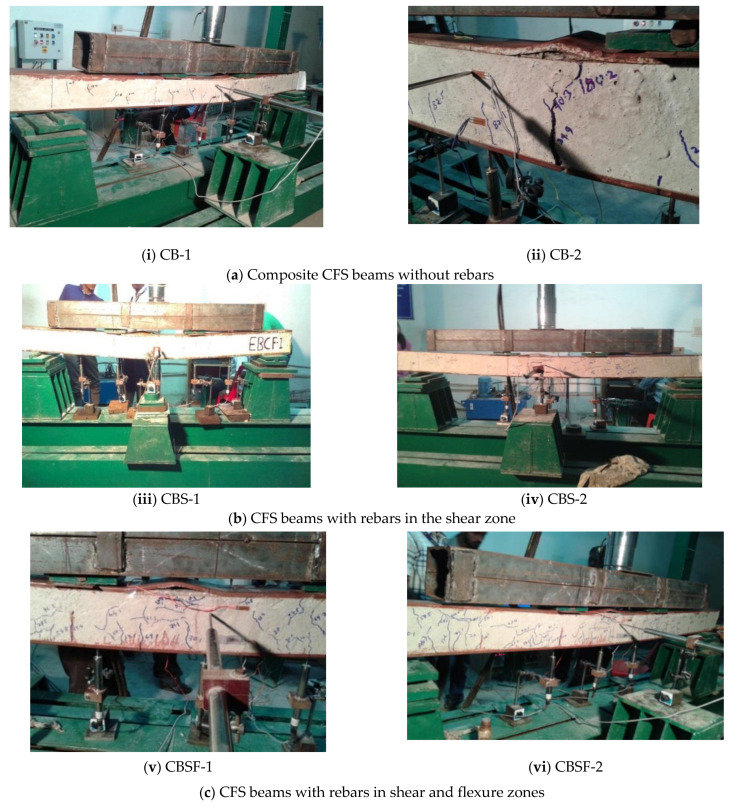
Failure of composite CFS beams.

All the beams with concrete-encased web (CB, CBS, and CBSF) failed as a result of flexural cracks in the concrete near the bottom flange propagating to the top. The majority of the cracks were found in the flexural zone (the region between the point loads), with a few appearing near the supports. The lips provided some refinement to the concrete and increased its load-carrying capacity (Figure 5).

### 4.2. Load–Deflection Relationships

**Figure 6:**
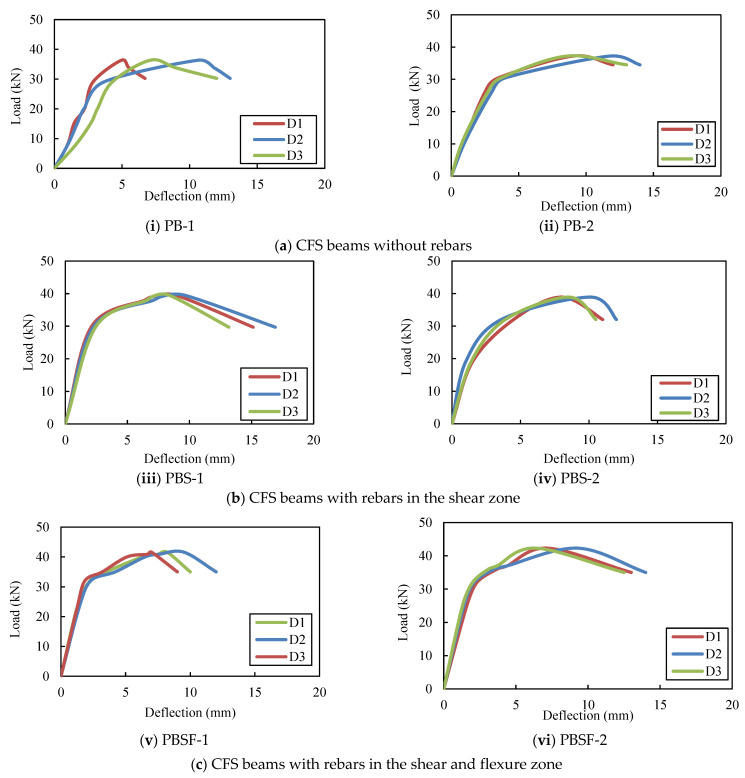
Load versus deflections of the plain CFS beams.

**Figure 7:**
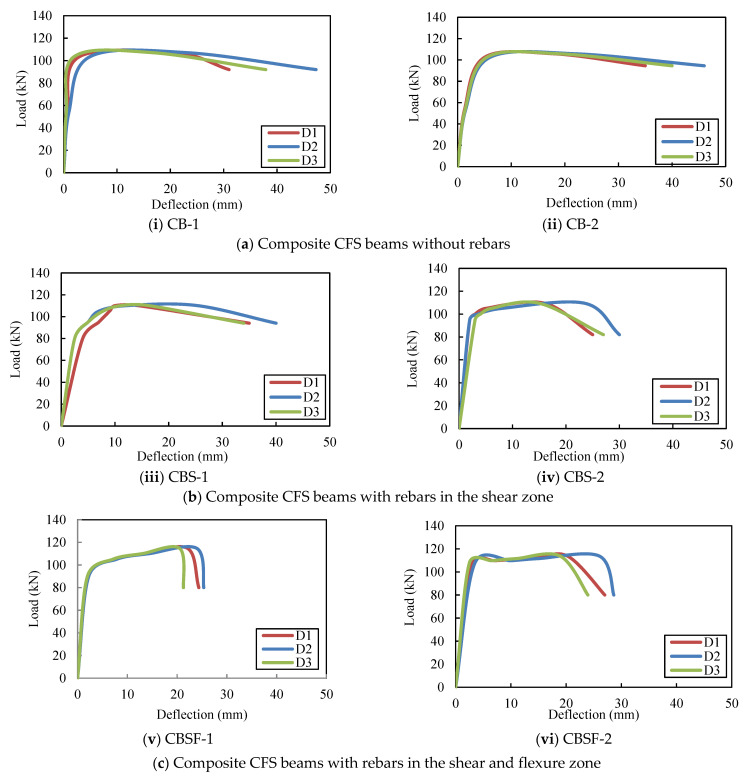
Load versus deflection curves of the composite CFS beams.

Up to the yield value, the load–deflection curves of the beams exhibit linear behaviour (Figure 6 and Figure 7). After the yield point, the variation was nonlinear until the ultimate stress, and the beams deflected excessively after reaching the ultimate stress point. The greatest deflection occurred in the middle of the span. Table 2 shows the load–deflection results. The deflections of the plain and composite CFS beams with and without diagonal rebars were within the IS 11384-1985 [41] and IS 800-2007 [42] serviceability codal limits, respectively. Plain CFS beams and composite CFS beams had deflections of less than 5.2 mm (Span/325 according to codes). The average ultimate loads for the plain beams, PB, PBS, and PBSF, were 37, 39, and 42 kN, respectively. Thus, the ultimate load-carrying capacity of the beams PBS and PBSF increased by 7% and 14%, respectively, when compared to the beams PB.

The average ultimate loads for the plain CFS beams CB, CBS, and CBSF, respectively, were 106 kN, 110 kN, and 114 kN. Thus, the ultimate load-carrying capacity of the beams CBS and CBSF increased by 3% and 7%, respectively, when compared to the CB beams.

**Figure 8:**
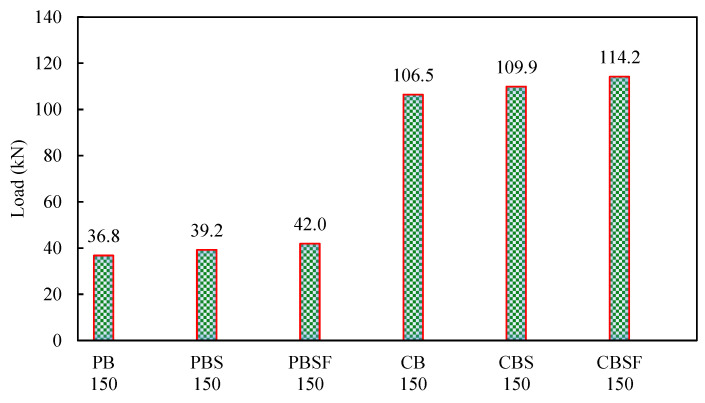
Comparison of the load capacities of the CFS beams.

The CBSF beam’s average ultimate load-carrying capacity was 2.7 times that of the PBSF beam. Figure 8 compares the load capacities of the CFS beams. As a result, CFS beams with fly ash concrete encased webs could be used to carry heavy loads in buildings while maintaining a low floor height.

### 4.3. Ductility of CFS Beams

**Figure 9:**
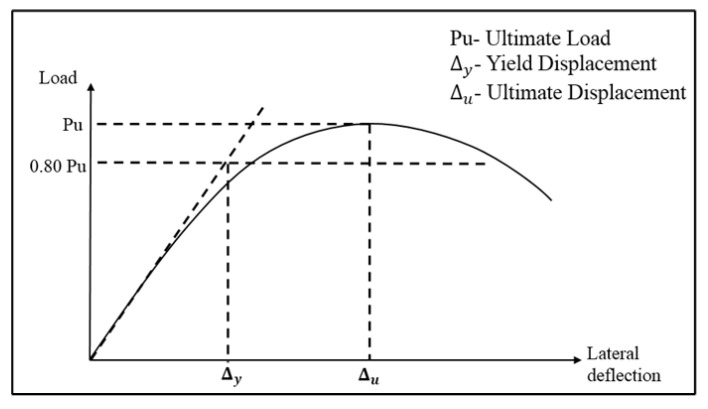
Evaluation of the yield and ultimate displacements [43].

As shown in Figure 9, the yield displacement of an equivalent elasto-plastic mechanism with reduced stiffness was calculated as the tangent stiffness at 80% of the ultimate load of the actual system [43]. The ultimate displacement was determined by taking the deflection corresponding to the ultimate load in the load versus displacement curve. The ductility values of all the tested specimens are shown in Table 3. The PBSF and CBSF beams had ductility factors of 3.2 and 8.1, respectively. By encasing the web in fly ash concrete, the ductility was increased by 153%.

The ductility values of the PBSF and PBS beams were 1.2 and 1.1 times higher, respectively, than those of the PB beams. The ductility values of the composite beams CBSF and CBS were 1.1 and 1.03 times greater than those of the CB beams, respectively. Because the composite CFS beams are ductile, they could be used in earthquake-resistant structures [44].

### 4.4. Load–Strain Relationship

**Figure 10:**
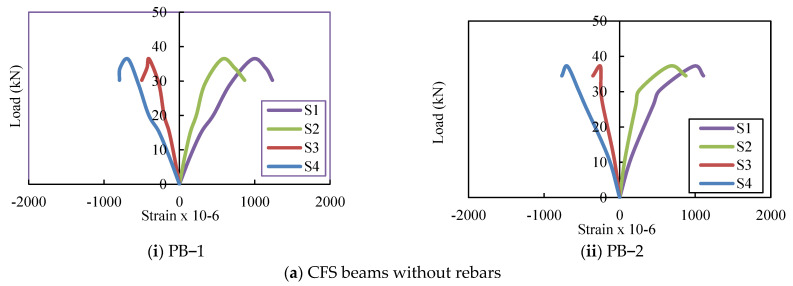

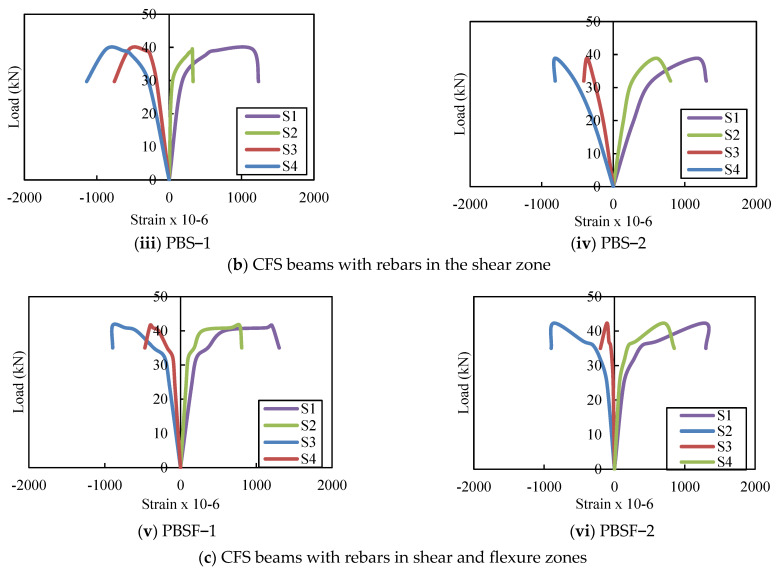
Load versus strain curves for plain CFS beams.

Figure 10 depicts the load–strain curves of the CFS beams. The strains measured at the ultimate load for PB-1 and 2 on the top and bottom flanges ranged from 970.3 to 978.9 and 681.1 to 691.3, respectively. The strains on the top and bottom flanges of PBS-1 and 2 ranged from 1148.4 to 1170.1 and 810.3 to 868.2, respectively, while the strains on the top and bottom flanges of PBSF 150-1 and 2 ranged from 1205.3 to 1289.4 and 863.6 to 894.6, respectively. The average ultimate compressive strains for plain CFS beams on the top flange ranged from 970.3 to 1289.4 and from 681.1 to 894.6 on the bottom.

**Figure 11:**
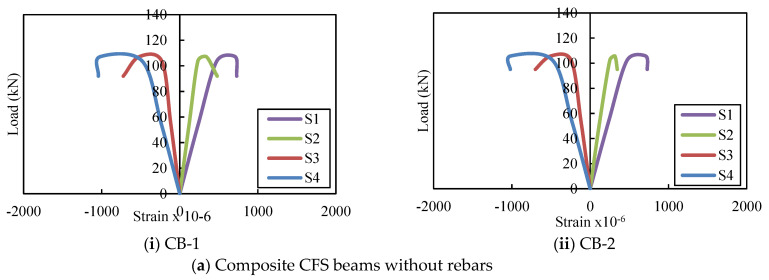

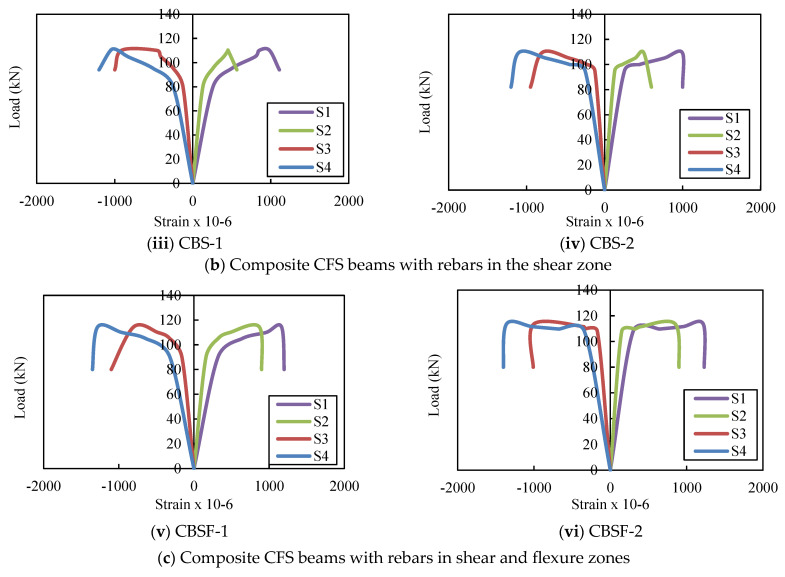
Load versus strain curves of the composite CFS beams.

Figure 11 depicts the load–strain behaviour of composite CFS beams. The average compressive strain on the upper flange of composite CFS beams with no rebars in the web (CB), rebars in the shear zone of the web (CBS), and rebars in the shear and flexure zones of the web (CBSF) was 0.03%, and the upper flange began to buckle. Maximum loads applied on average were 82 kN, 88 kN, and 90 kN, respectively. The corresponding ultimate strains and loads were 967με, 1074με, and 1319με, respectively, and 106, 110, and 114 kN.

### 4.5. Moment vs. Curvature Relationships

The curvature (∅) of the plain and composite CFS beams was calculated using Equation (1):

(1) $$\emptyset =\frac{\left(\varepsilon_{c}+\varepsilon_{t}\right)}{d}$$

where,

*ε_c_*—compressive strain at the top flange,
*ε_t_*—tensile strain at the bottom flange,
*d*—depth of the beam.

**Figure 12:**
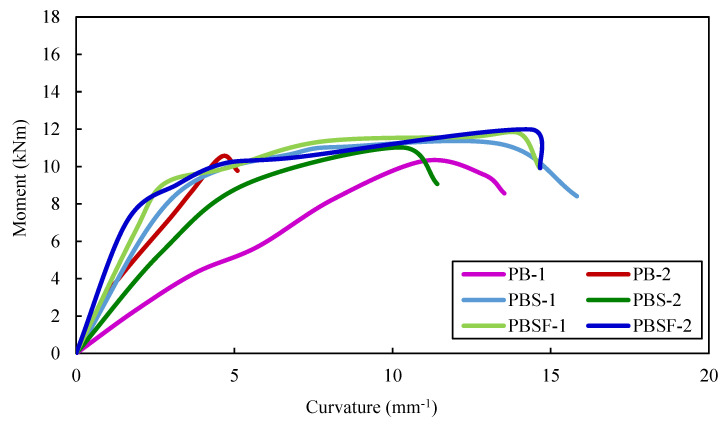
Moment–curvature curves of the plain CFS beams.

The moment–curvature curves of the plain CFS beams are shown in Figure 12. The curves show that CFS beams with rebars in the shear and flexure zones in the web, as well as composite CFS beams with rebars in the shear and flexure zones in the web (PBSF-1 and 2 and CBSF-1 and 2), had higher moment capacities. CFS beams PB, PBS, and PBSF had average ultimate moment capacities of 10.4 kNm, 11.1 kNm, and 11.9 kNm, respectively.

The average ultimate moment capacities of composite CFS beams (CB, CBS, and CBSF beams) were 30.2 kNm, 31.1 kNm, and 32.3 kNm, respectively.

**Figure 13:**
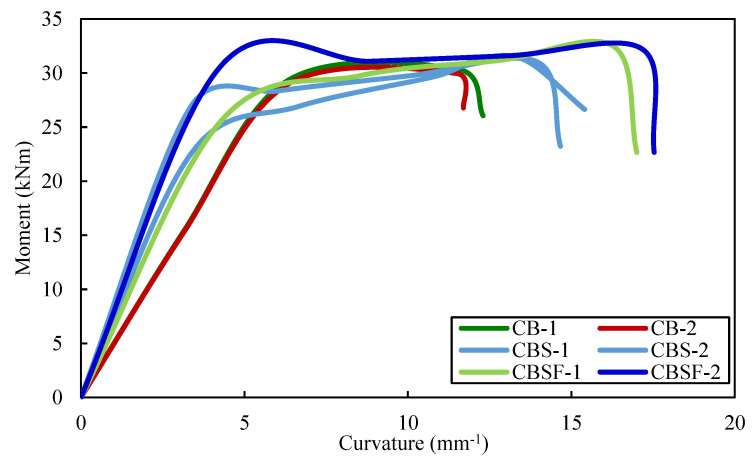
Moment–curvature curves of the composite CFS beams.

The moment–curvature curves of the composite CFS beams are shown in Figure 13. The average ultimate moment capacity of the beam CB was 2.9 times greater than that of the beam PB, 2.8 times greater than that of the beam PBS, and 2.7 times greater than that of the beam CBSF. Thus, encasing the web of plain CFS beams with fly ash concrete increased the moment capacity by approximately 2.8 times over plain CFS beams.

Encasing the webs in concrete increases the curvature of the composite beam CB 1.5 times more than the beam PB; it increases the curvature of the beam CBS 1.2 times more than the beam PBS and it increases the curvature of the beam CBSF 1.2 times more than the beam PBSF.

Because the composite CFS beams demonstrated strong moment–curvature relationships, they could be used in earthquake-resistant structures.

### 4.6. Lateral Buckling Resistance

**Figure 14:**
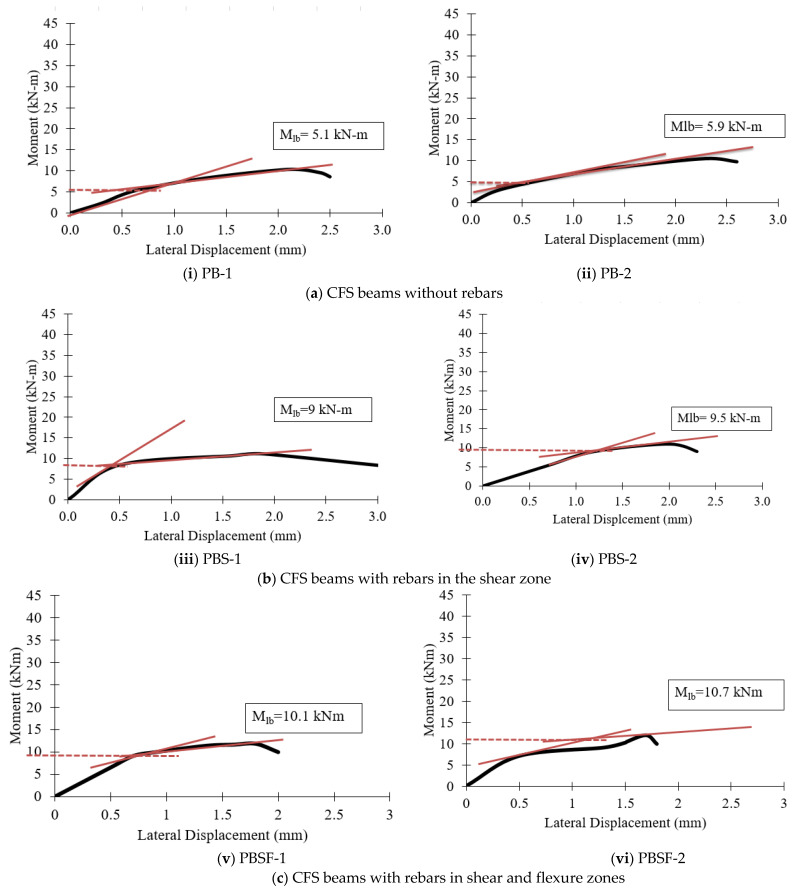
Moment–lateral displacement curves of the plain CFS beams.

**Figure 15:**
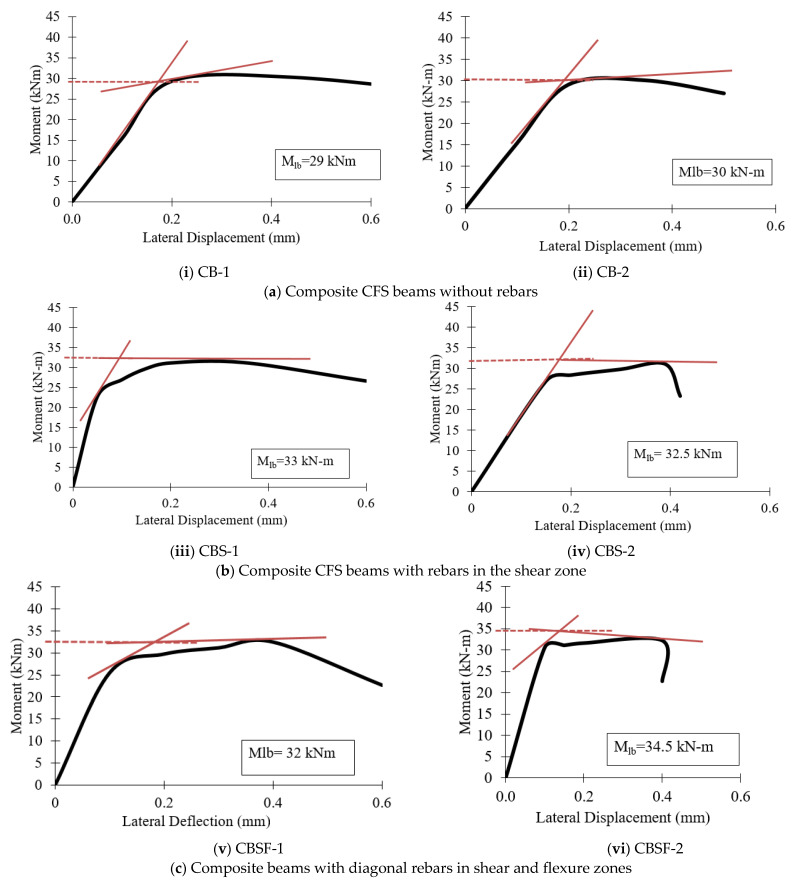
Moment–lateral displacement curves of the composite beams.

The values for lateral buckling resistance were determined at the intersection of the drawn tangents, the point where the curve became nonlinear, and at the point of ultimate loading. The lateral moment resistance of the beams was determined using the “knee joint” intersection method [45]. When a “knee” shape was observed, the values of lateral buckling resistance (Mlb) for all specimens were determined. Two tangents were drawn for each plot, and their intersection was used to calculate the Mlb value. Figure 14 and Figure 15 show the moment–lateral displacement curves of plain CFS and composite CFS beams, respectively.

**Figure 16:**
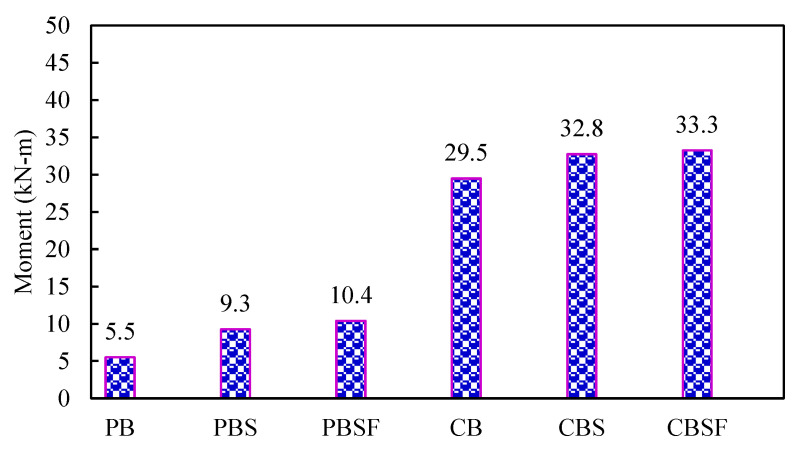
Comparison of the lateral buckling moments of the beams.

Figure 16 shows that plain CFS beams with diagonal rebars in the shear and flexure zone (PBSF) and composite CFS beams with diagonal rebars in the shear and flexure zone (CBSF) performed well and had higher lateral buckling moments.

## 5. Finite Element Analysis of CFS Beams

### 5.1. Details of the Model

**Figure 17:**
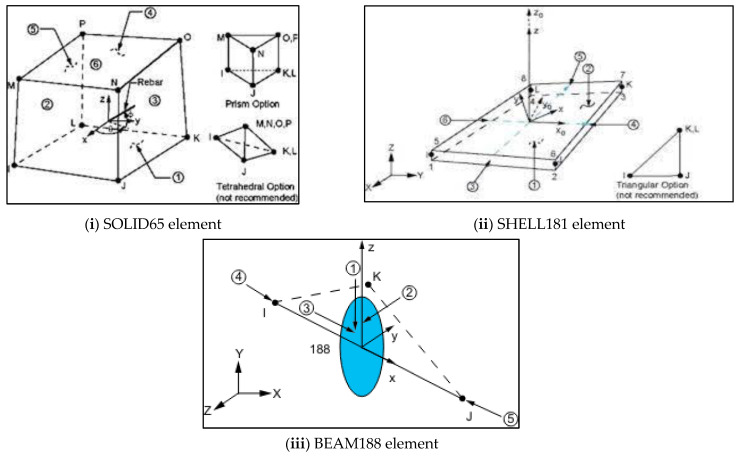
Elements used in the finite element model.

**Figure 18:**
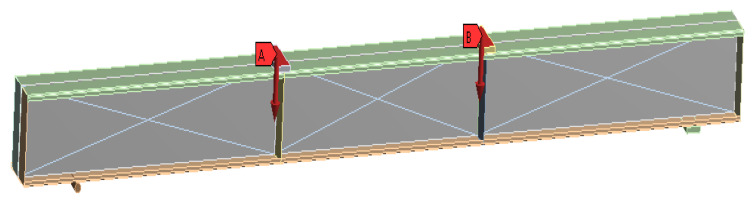
Loading applied on the FE model.

**Figure 19:**
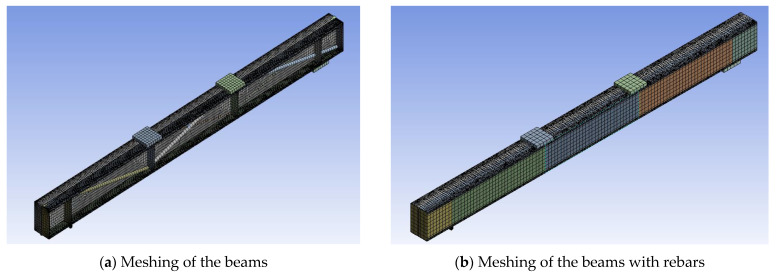
Meshing of beams used in the FE model.

Figure 17, Figure 18 and Figure 19 show the details of finite element modelling of plain and composite CFS beams using ANSYS software 2022-R1 [46]. The modelling steps include the selection of element type, material property assignment, modelling, and element meshing.

To replicate the actual physical setup used in the experiments, the full geometry was modelled. The I-beam structure was constrained by the addition of a rigid roller and a support plate (flat member) at the bottom of the beams. One end of the beams was restrained against displacement in both the horizontal and vertical axes, while the other end was restrained against displacement in both the horizontal and vertical axes.

The SOLID65 element, a 3D hexahedral element with eight nodes, was used to mesh the concrete. The SHELL181 element, a 2D element with four nodes and six degrees of freedom for each node, was used to mesh the CFS section. For mesh generation, the collapsing nodal triangular option was used. BEAM 188 elements were used to mesh the welded rebars to the web. The model considered the relationship between stress and strain of concrete in compression to be nonlinear [47]. Except for the loading plate, which was defined as rigid, the entire beam was modelled as deformable [48]. The mesh size was determined using mesh convergence studies in relation to the experimental results. The contact between concrete and CFS was modelled using CONTA174 and TARGE170 finite elements with a friction coefficient of 0.45 [49]. In the simulation, the displacement rate was set to 1 mm/min.

### 5.2. Deformed Shapes of CFS Beams

**Figure 20:**
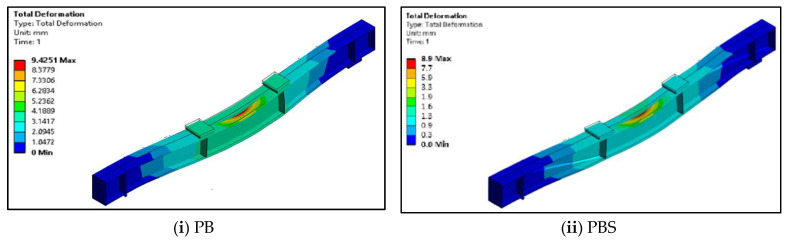

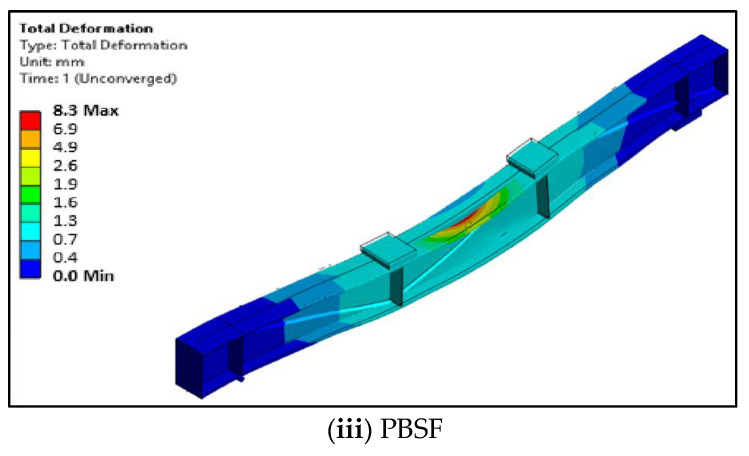
Deflected shapes of the plain CFS beams.

**Figure 21:**
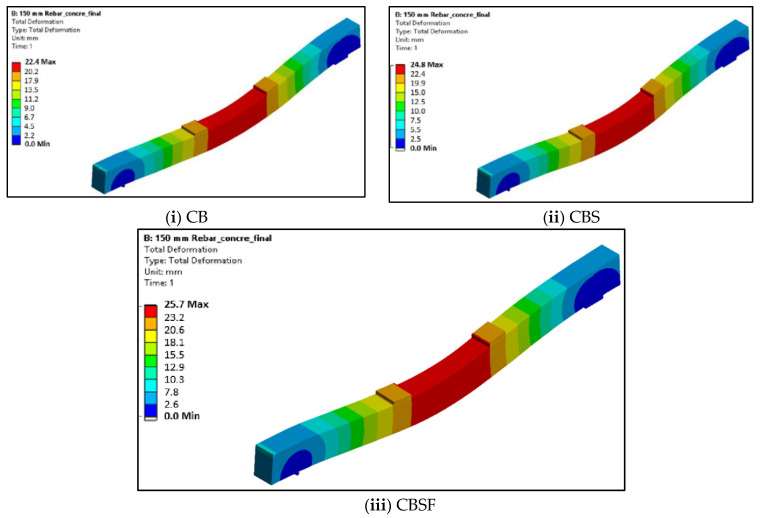
Deflected shapes of the composite CFS beams.

Figure 20 and Figure 21 show the geometries of the deformed shapes of the plain CFS and composite CFS beams. The ultimate loads obtained from the FEA for CFS beams PB, PBS, and PBSF were 32 kN, 35.4 kN, and 36.1 kN, respectively, with corresponding deformations of 9.4 mm, 8.9 mm, and 8.3 mm.

The ultimate loads obtained from the FEA for composite beams CB, CBS, and CBSF were 100.9 kN, 105 kN, and 108.8 kN, respectively, with corresponding deformations of 22.4 mm, 24.8 mm, and 25.7 mm.

### 5.3. Comparison of the Experimental Results with the FE Results

**Figure 22:**
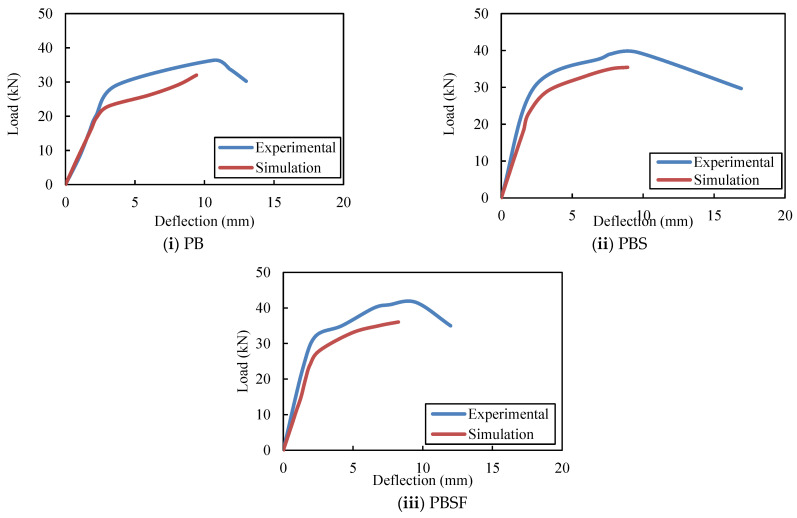
ANSYS and experimental deflections of the CFS beams.

Figure 22 depicts the load–deflection results obtained from experiments and FEA for CFS beams without rebars in the web (PB), with rebars in the shear zone (PBS), and with rebars in both the shear and flexure zones (PBSF). The variations in strengths between the FE models and experiments are found to be between 1% and 16%, and the deviations in deformations between the FE models and experiments are found to be between 8% and 19%.

**Figure 23:**
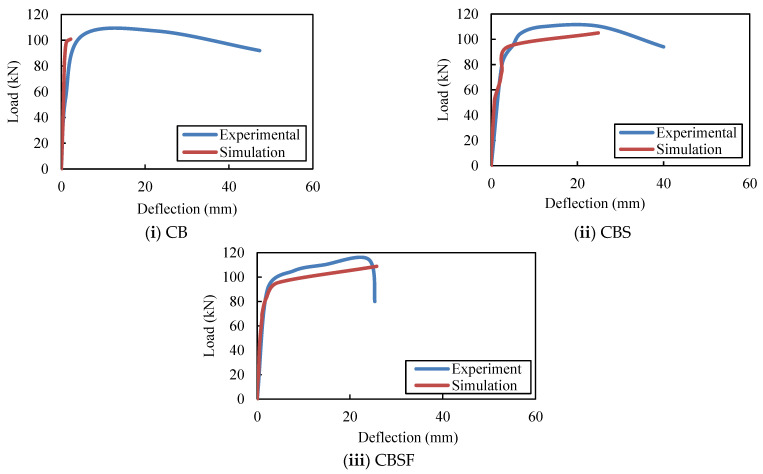
ANSYS and experimental deflections of the composite CFS beams.

Figure 23 depicts the experimental and FEA load–deflection results for composite CFS beams without rebars in the web (CB), with rebars in the shear zone (CBS), and with rebars in both the shear and flexure zones (CBSF). The variations in strengths between the FE models and experiments are found to be between 4% and 6%, and the deviations in deflections between the FE models and experiments are found to be between 1% and 14%.

### 5.4. Performance Assessment of the Composite CFS Beams

CFSB beams have a two times higher ultimate moment carrying capacity than PBSF beams. As a result, CBSF beams could be installed in buildings with lower floor heights. CBSF beams have improved elastoplastic properties and ductility which is 2.5 times that of PBSF beams. The CBSF beams also had a high moment–curvature. As a result, CBSF beams could be used in earthquake-prone buildings (Table 4).

## 6. Conclusions

The following conclusions were reached after conducting experimental and numerical investigations on plain and composite CFS beams, both having diagonal rebars in the web:

1. When compared to plain beams without diagonal rebars, the ultimate load-carrying capacity of plain beams with diagonal rebars in the shear and flexure zones (PBSF) increased by 14% (PB).
2. The addition of rebars to the PBSF beams’ shear and flexure zones increased ductility by 1.2 times that of the PB beam.
3. When compared to PB beams, the lateral buckling capacity of PBSF beams increased by 66%.
4. When compared to the composite beam without diagonal rebars, the ultimate load-carrying capacity of the composite beams with diagonal rebars in the shear and flexure zones (CBSF) increased by 7% (CB).
5. The addition of rebars to the CBSF beams’ shear and flexure zones increased its ductility by 1.1 times that of the CB beam.
6. When compared to CB beams, the lateral buckling capacity of CBSF beams increased by 10%.
7. The beam CBSF’s ultimate load-carrying capacity was 2.7 times that of the beam PBSF.
8. The finite element analysis results were in good agreement with the experimental investigation.
9. Finally, it can be concluded that using the composite CFS beam, the building’s floor height can be reduced, earthquake-resistant structures can be built, and significant sustainability in the industry can be practised.

## Figures and Tables

**Table 1:** Details of the CFS beams.

Designation	Description
PB-1 and 2	Plain beams without diagonal rebars
PBS-1 and 2	Plain beams with rebars in the flexure zone
PBSF-1 and 2	Plain beams with rebars in shear and flexure zones
CB-1 and 2	Composite beams without diagonal rebars
CBS-1 and 2	Composite beams with rebars in the flexure zone
CBSF-1 and 2	Composite beams with rebars in shear and flexure zones

Note: The first two letters indicate whether the CFS beam has a plain web or is encased in concrete. The third and fourth letters indicate whether the beam has rebars in the shear zone or the shear and flexure zone.

**Table 2:** Load–deflection results.

Specification of CFS Beam Specimens	Ultimate Load (kN)	Deflection at Ultimate Load (mm)	Deflection at Yield Load (mm)
PB-1	36.4	10.7	4.3
PB-2	37.2	11.7	3.7
PBS-1	39.5	9.6	2.5
PBS -2	38.9	10.1	3.1
PBSF-1	41.6	9.5	2.3
PBSF-2	42.3	9.3	2.0
CB-1	107.1	23.0	2.5
CB-2	105.8	22.0	3.2
CBS-1	110.3	25.0	2.5
CBS-2	109.5	23.5	2.9
CBSF-1	114.6	24.0	2.0
CBSF-2	113.7	26.0	2.3

**Table 3:** Ductility values of CFS beams.

Sl. No.	Beam Number	Deflection at Yield Load (mm)	Deflection at Ultimate Load (mm)	Displacement Ductility Ratio	Average Displacement Ductility Ratio
1	PB-1	4	10.7	2.7	2.6
2	PB-2	4.6	11.7	2.5	
3	PBS-1	3.3	9.6	2.9	2.9
4	PBS-2	3.6	10.1	2.8	
5	PBSF-1	3	9.5	3.2	3.2
6	PBSF-2	2.8	9.3	3.3	
7	CB-1	3	23	7.7	7.5
8	CB-2	3	22	7.3	
9	CBS-1	3.2	25	7.8	7.7
10	CBS-2	3.1	23.5	7.6	
11	CBSF-1	2.9	24	8.3	8.1
12	CBSF-2	3.25	26	8.0	

**Table 4:** Performance assessment of the beams investigated in this study.

Beam	Ult. Moment (kNm)	Deflection at Max Load (mm)	Ultimate Compressive Strain (εc) %	Ultimate Tensile Strain (εt) %	Curvature at the Ultimate Moment (m^−1^)	Ductility Ratio
PB-1	36.4	10.7	970.3	681.1	11.0	2.7
PB-2	37.2	11.7	978.9	691.3	9.6	2.5
PBS-1	39.5	9.6	1148.4	868.2	13.4	2.9
PBS-2	38.9	10.1	1170.1	810.3	10.3	2.8
PBSF-1	41.6	9.5	1205.3	894.6	14.0	3.2
PBSF-2	42.3	9.3	1289.4	863.6	14.4	3.3
CB-1	107.1	23.0	710.6	1020.4	11.5	7.7
CB-2	105.8	22.0	708.4	1015.6	11.4	7.3
CBS-1	110.3	25.0	981.3	1047.7	13.5	7.8
CBS-2	109.5	23.5	994.7	1098.6	14.0	7.6
CBSF-1	114.6	24.0	1164.1	1279.5	16.3	8.3
CBSF-2	113.7	26.0	1216.7	1358.6	17.2	8.0

## Data Availability

The data presented in this study are available on request from the corresponding author.
